# Impact of Direct-Acting Antivirals on Extrahepatic Manifestations in Chronic Hepatitis C: A Narrative Review with a Hermeneutic Approach

**DOI:** 10.3390/healthcare13161953

**Published:** 2025-08-09

**Authors:** Alexia Anastasia Stefania Balta, Mariana Daniela Ignat, Raisa Eloise Barbu, Caterina Dumitru, Diana Sabina Radaschin, Valentin Bulza, Silvia Aura Mateescu Costin, Catalin Pleșea-Condratovici, Liliana Baroiu

**Affiliations:** 1 Doctoral School of Biomedical Sciences, ‘Dunarea de Jos’ University, 800008 Galati, Romania; alexiaanastasia1998@yahoo.com (A.A.S.B.); valibulza@gmail.com (V.B.); silviaaura79@gmail.com (S.A.M.C.); 2 Faculty of Medicine and Pharmacy, ‘Dunarea de Jos’ University, 800008 Galati, Romania; dumitrukati@gmail.com (C.D.); dianaradaschin@yahoo.com (D.S.R.); dr.catalinpleseauniv@gmail.com (C.P.-C.); lilibaroiu@yahoo.com (L.B.); 3 ‘Sf. Apostol Andrei’ Clinical Emergency County Hospital, 800578 Galati, Romania; 4 ‘Sf. Cuv. Parascheva’ Clinical Hospital of Infectious Diseases, 800179 Galati, Romania; 5 ‘Sf. Ioan’ Clinical Hospital for Children, 800487 Galati, Romania; 6 Multidisciplinary Integrated Centre of Dermatological Interface Research (MICDIR), ‘Dunarea de Jos’ University, 800385 Galati, Romania; 7 Galati Railways General Hospital, 800225 Galati, Romania

**Keywords:** hepatitis C virus, extrahepatic manifestations, cryoglobulinemia, DAAs, SVR

## Abstract

**Background/Objectives**: Chronic hepatitis C virus (HCV) infection is associated with a wide spectrum of extrahepatic manifestations, involving the immune, dermatologic, endocrine, vascular, and neuropsychiatric systems. Among these, mixed cryoglobulinemic vasculitis (CryoVas) remains one of the most clinically relevant complications. This work aims to provide a structured overview of HCV-related extrahepatic conditions and to analyze the clinical and virological outcomes of direct-acting antivirals (DAAs) in CryoVas patients. **Methods**: We first categorized and reviewed extrahepatic manifestations of HCV across five major domains: immune, inflammatory/metabolic/vascular, dermatological, thyroid, and neuropsychiatric. Subsequently, we conducted a comparative analysis of five clinical studies evaluating the impact of DAA therapy in patients with CryoVas. Data on demographics, clinical symptoms, treatment regimens, sustained virological response, and clinical response were extracted and summarized. **Results**: HCV was found to be associated with numerous extrahepatic conditions, including mixed cryoglobulinemia, non-Hodgkin lymphoma, autoimmune thyroiditis, insulin resistance, and neurocognitive symptoms. In the CryoVas subgroup analysis, virological response rates were uniformly high (88.9–100%), but clinical remission varied significantly. Complete response ranged from 39% to 90%, highlighting a discrepancy between viral eradication and extrahepatic symptom resolution. These findings underscore the need for individualized follow-up and further investigation into persistent immunological dysfunction post-sustained virological response (SVR). However, clinical outcomes were more variable: complete response (CR) varied between 39% and 90%, partial response (PR) ranged from 4% to 42%, and no response (NR) was reported in 0% to 40% of cases. Although significant improvement in key manifestations such as purpura, arthralgia, and neuropathy was frequently observed, a subset of patients continued to exhibit residual or refractory symptoms despite achieving SVR. **Conclusions**: HCV infection exerts multisystemic effects that extend beyond liver pathology. While DAAs offer near-universal virological clearance, the heterogeneous clinical response in CryoVas underscores the need for closer monitoring of extrahepatic outcomes. Future research should assess whether combining DAAs with immunomodulatory strategies can improve symptom control and long-term outcomes in patients with severe or refractory CryoVas.

## 1. Introduction

The World Health Organization has estimated that hepatitis C caused approximately 242,000 deaths in 2022. An estimated 1 million new HCV infections happen yearly, with 50 million people living with chronic infection. HCV infections are known to put patients at risk for major side effects like hepatocellular cancer and liver cirrhosis. DAAs cure approximately 95% of hepatitis C cases [1]. DAAs have significantly improved treatment outcomes and prognosis for chronic HCV patients [2].

Up to two-thirds of HCV patients experience symptoms of extrahepatic manifestations, including autoimmune and rheumatic conditions (myalgia, arthralgia, sicca syndrome, various vasculitis’s) (Table 1). These symptoms can be more severe than the progression of the liver infection itself [2]. Chronic HCV infection is linked to many non-liver-related health issues [3]. One unique type of kidney illness that is unmistakably linked to HCV infection is cryoglobulinemic nephritis (CN). According to estimates, CN develops in approximately 0.5 percent of individuals infected with HCV and represents a significant factor influencing prognosis [3]. These manifestations can affect various systems and may significantly impact patient prognosis and quality of life. The table below provides a system-based categorization of these conditions, based on current clinical observations and literature (Table 1) [4]. Soon after HCV was discovered, reports of autoimmune or lymphoproliferative illnesses linked to HCV infection started to surface. These diseases ranged from benign mixed cryoglobulinemia to overt lymphomas [5,6].

HCV infection has been linked to increased mortality risk from extrahepatic consequences [7,8,9]. Patients with HCV had twice the mortality rate of those without HCV because of the presence of serum hepatitis C virus RNA (HCV RNA) [7,10].

**Figure 1:**
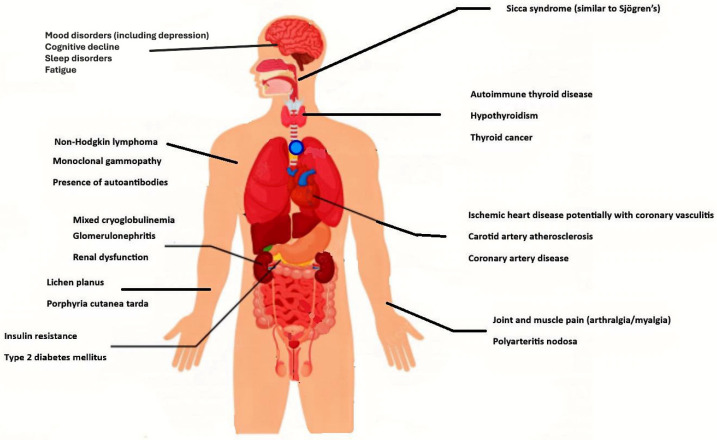
Extrahepatic manifestations associated with HCV infection. Image source: https://quizlet.com/370092963/body-internal-organs-flash-cards/ (accessed on 16 July 2025) [11].

Figure 1 illustrates the anatomical tropism, distribution, and specific types of extrahepatic manifestations associated with chronic HCV infection, offering a visual overview of the systemic involvement characteristic of the disease. Based on a synthesis of clinical studies, the most frequently reported extrahepatic manifestations (EHMs) include:

The graphical representation emphasizes the systemic nature of chronic HCV infection and highlights the need for a multidisciplinary diagnostic and therapeutic approach, especially in the context of initiating antiviral therapy with direct-acting antivirals.

A study of 22,576 HCV-infected patients in Canada found that SVR with DAAs was associated with a lower risk of chronic kidney disease, major adverse cardiac events, stroke, and neurocognitive impairment compared with no treatment. No significant differences were observed in the risk of developing type 2 diabetes [12].

Also, a meta-analysis of clinical trials [3] found that HCV patients with SVR after DAA therapy had a lower risk than those who did not respond to treatment of developing B-cell non-Hodgkin lymphoma and cryoglobulinemic vasculitis. The same meta-analysis noted that SVR with DAAs reduces the risk of cardiovascular disease, acute coronary syndrome, type 2 diabetes, and improves atherosclerosis, increasing the quality of life and survival of patients with HCV and associated extrahepatic manifestations.

Another meta-analysis of clinical trials [13] reported studies suggesting a reduced risk of extrahepatic cancers after DAA therapy, while others showed no significant change. These findings emphasize the need for future studies with longer post-SVR monitoring periods to draw solid conclusions regarding the evolution of neoplasias after SVR and underscore the importance of careful monitoring of these patients.

## 2. Materials and Methods

A broad literature search was conducted using PubMed, Google Scholar, and ResearchGate to identify clinical studies and meta-analyses published between 2016 and 2024. The search focused on key terms such as hepatitis C virus, treatment, antiviral therapies, clinical studies, and extrahepatic complications.

Sources were selected based on their clinical relevance and thematic alignment with the aim of this paper, with particular attention given to studies reporting on vasculitis and other systemic extrahepatic manifestations in patients with chronic HCV infection.

Priority was given to studies involving adult patients who were informed and provided informed consent, with active chronic HCV infection confirmed by detectable viral RNA, and clinically evident vasculitis affecting one or more systems, such as the skin, joints, kidneys, or peripheral nerves. Patients were considered regardless of HCV genotype, prior antiviral treatment, or treatment response.

Studies were excluded if they involved patients under the age of 18, inactive cryoglobulinemic vasculitis, co-infection with human immunodeficiency virus (HIV) or hepatitis B virus, or decompensated liver cirrhosis.

The studies were analyzed using the AMSTAR checklist for quality criteria and valid conclusions [14].

**Figure 2:**
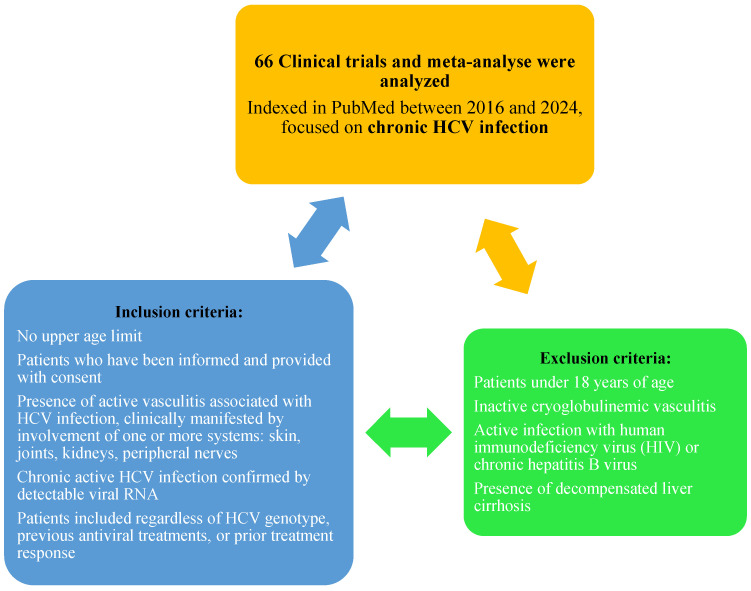
Flow diagram of literature search [16].

Out of a total of 66 articles and studies reviewed, 9 studies met the inclusion criteria and were selected for analysis (Figure 2). All 66 studies analyzed, including the 9 selected, were prospective or retrospective observational clinical studies on patients with HCV infection and extrahepatic manifestations who received antiviral treatment with DAAs.

It is important to note that the available data were insufficient for a conventional systematic review or meta-analysis. Rather than aiming for exhaustive or systematic coverage, this work adopts a hermeneutic approach to interpret and contextualize the existing clinical evidence. Grounded in the concept of *verstehen*—interpretive understanding—it seeks to generate nuanced insights through critical reflection on individual studies within the broader medical discourse [15].

## 3. Results

### 3.1. Mixed Cryoglobulinemia (MC)

The hepatitis C virus is present in up to 90% of patients with mixed cryoglobulinemia. Polyclonal IgG and either monoclonal (type II) or polyclonal (type III) IgM with rheumatoid factor activity are involved in this syndrome [17,18]. About 15% of individuals with chronic HCV have clinically evident cryoglobulinemic vasculitis (CryoVas), whereas 40–60% of patients have circulating mixed cryoglobulins [19,20].

Between 45 and 60 percent of treated patients with cryoglobulinemia experienced a complete immunologic response. Combination therapy with daclatasvir and sofosbuvir has been shown to effectively cure regulatory T-cell deficits and increase Th17 cells, follicular helper T cells, and IgM+ CD21 low memory B cells in individuals receiving treatment. These results suggest that mixed cryoglobulinemia can be completely and sustainably cleared [19].

The study by Saadoun et al. shows that a rapid clinical and virologic response can be achieved through a combination of antiviral therapy, including interferon (IFN) and ribavirin (RIBA) together with sofosbuvir and daclatasvir. Up to ninety percent of patients diagnosed with CryoVas experienced a complete clinical response. Tolerance was satisfactory, and no adverse reactions occurred [19]. Patients with HCV-CryoVas who present with severe disease symptoms such as membranoproliferative glomerulonephritis, severe neuropathy, or other serious complications are typically prescribed immunosuppressive agents. In the study by Saadoun et al., it was observed that only 4.8% of patients required combination therapy, including antivirals, rituximab and glucocorticosteroids [19] (Table 2).

The clinical expression of the disease can range from mild symptoms to severe, life-threatening complications. Palpable purpura is the primary skin symptom. Chronic skin ulcers, acrocyanosis, Raynaud’s phenomenon, and digital ulcerations may also be present [21].

CryoVas is mostly caused by hepatitis C virus infection, and patients with mixed CryoVas that are HCV-positive, mixed CryoVas that are HCV-negative, and type I CryoVas have respective ten-year survival rates of 63 percent, 65 percent, and 87 percent [21]. In 70 percent of cases, patients present with arthralgia affecting large peripheral joints, which is rarely associated with arthritis. Distal sensory or sensorimotor polyneuropathy, often accompanied by painful and asymmetric paresthesias, is the most frequently reported neurological manifestation [2,22].

Since viral clearance is directly linked to clinical remission of vasculitis, treating HCV-related CryoVas is a difficult endeavor [23]. Thus, achieving a sustained virologic response in these individuals is the main objective [24]. Another recent study by Gragnani et al. reports that 4.5% of patients required this combination therapy [25].

An important objective in the treatment of HCV-CryoVas is the clearance of cryoglobulins. Indeed, in some HCV patients, vasculitis occurs despite achieving SVR. This is usually associated with persistent cryoglobulinemia and B-cell lymphoma [26].

Current evidence highlights the superior efficacy of DAAs in achieving SVR and significant clinical remission in patients with HCV-associated mixed cryoglobulinemia. Studies report CryoVas remission rates as high as 95.2% and improved long-term outcomes, particularly when non-antiviral immunosuppressive therapies were minimized. Although a subset of patients remains cryoglobulin-positive or experiences persistent symptoms post-SVR, DAAs consistently outperform older regimens such as pegylated interferon plus ribavirin or early triple therapies. These findings underscore the critical role of early and targeted antiviral therapy in mitigating the extrahepatic complications of chronic HCV infection (Table 2).

**Table 2:** Summary of clinical outcomes in HCV-related cryoglobulinemic vasculitis.

Study	Key Findings	Reference
Cacoub et al.	CryoVas remission: 95.2%; severe neuropathy/vasculitis = 3× less likely to achieve full clinical response	[27]
DAA era	CryoVas prevalence: 14.3%; better antiviral response when non-antiviral treatment was reduced; the study by Cacoub et al. reported long-term response: 46.9%	[27]
French cohort	Use of non-antiviral treatment decreased from 43% (IFN-RBV era) to 4.8% (sofosbuvir + daclatasvir era)	[28,29]
Mortality rates	25% at 5 years; 40% at 10 years in HCV-CryoVas patients	[21,30]
DAA-treated patients	50–61% remained cryoglobulin-positive despite SVR	[31,32]
Lauletta et al.	Significant clinical improvement with DAAs; fewer and milder adverse reactions; 27.3% had persistent or worsened symptoms despite SVR12	[33]
Comparison with older therapies	DAAs yielded better outcomes vs. pegylated IFN + RBV and earlier triple therapy with first-generation protease inhibitors	[29,34]
Small pilot studies (IFN + RBV)	IFN + RBV improved outcomes in chronic HCV + mixed cryoglobulinemia	[34,35]
DAA in IFN non-responders	Among IFN-α monotherapy non-responders, 19–54% achieved SVR and mixed cryoglobulinemia remission with DAAs	[36]

Major predictors of CryoVas in HCV patients include advanced age, type II mixed cryoglobulinemia, prolonged infection, and liver or blood clonal expansions of B cells. Renal symptoms and age over 60 are known as more severe indicators. Studies associate kidney damage with a five-year survival rate ranging from 90 percent to 50 percent. Other causes of death include cancer, infectious illnesses, cardiovascular disease, and liver disease [37]. HCV-CryoVas can cause either progressive damage (renal involvement) or acute complications (intestinal, cardiac, central nervous system, pulmonary hemorrhage), which are life-threatening and may result in fatal outcomes, with mortality rates ranging from 20 percent to 80 percent [38,39].

Studies show that DAA therapy clears HCV at similar rates in patients with and without mixed cryoglobulinemia. HCV clearance through DAA therapy in patients with MC varies from 88.9 percent to 100 percent, according to the key studies included in Table 3 [19,25,27,32,33,40,41,42,43].

A comparative analysis of clinical studies (Table 3 [19,25,27,32,33,40,41,42,43]) evaluating direct-acting antivirals for HCV-related cryoglobulinemic vasculitis demonstrates consistently high sustained virological response rates, typically exceeding 90%. The table presents an integrated analysis of nine clinical studies (2016–2020) investigating the efficacy of direct-acting antivirals in patients with HCV infection and associated mixed cryoglobulinemia. These studies, both prospective and retrospective in design, enrolled a total of 486 patients across various age groups. Most studies demonstrated high SVR rates (88.9–100%) across different DAA combinations (e.g., SOF-based, 3D, IFN-free), regardless of symptom severity or comorbidity profiles. Clinical manifestations such as purpura, arthralgia, polyneuropathy, and renal involvement were variably present, with purpura and arthralgia appearing most frequently (up to 83.3% and 75.6%, respectively).

Despite the excellent virological efficacy of DAAs, clinical response was heterogeneous. Complete clinical response ranged from 39% to 90%, partial response between 4% and 42%, and no response varied from 0% to 40%. Notably, retrospective studies reported a broader variability in clinical outcomes compared to prospective designs, possibly due to underlying selection and reporting biases. DAAs demonstrate consistently high SVR rates in patients with HCV-related cryoglobulinemic vasculitis, irrespective of the specific regimen used or study design. Clinical response remains variable, indicating that virological clearance does not always correspond to full remission of extrahepatic symptoms, suggesting possible irreversible immune-mediated damage. Future studies should focus on predictors of clinical remission, standardizing outcome measures, and integrating immunological biomarkers to better correlate SVR with symptom resolution.

This discrepancy between viral clearance and clinical remission suggests that persistent immune-mediated mechanisms may continue despite SVR. These findings highlight the need for adjunctive therapeutic strategies, especially in patients with refractory extrahepatic manifestations.

### 3.2. Non-Hodgkin’s Lymphoma (NHL) and B-Cell Non-Hodgkin’s Lymphoma (B-NHL)

NHL is a group of malignant lymphoproliferative disorders originating from lymphoid cells. It includes many subtypes, each with its own clinical, epidemiological, etiological, histological, immunopathological, and genetic characteristics [44]. Exposure to radiation, pesticides, obesity, benzene, smoking, and certain viral and bacterial infections are among the numerous risk factors for NHL [45,46,47]. HCV is known to have lymphotropic properties, and research has shown that it stimulates both T cells and B cells. HCV RNA and viral proteins have been found in lymphoma biopsy samples from patients with HCV-linked lymphoma [48,49]. The general population in Egypt exhibits the highest documented prevalence of HCV infection, exceeding 20%. In contrast, moderately elevated rates, ranging between 5% and 10%, have been reported in countries such as Italy and Japan. In the majority of other regions—including South Korea, Northern European countries, the United States, Australia, and Canada—the prevalence remains relatively low, typically under 5% [50,51].

A systematic review comprising 48 studies and a total of 5542 patients reported an average prevalence of hepatitis C virus infection of 13% (95% confidence interval: 12–14%) among individuals diagnosed with B-cell non-Hodgkin lymphoma. Notably higher prevalence rates were observed in specific regions, such as Italy (20%) and Japan (14%) [49].

This implies that HCV proteins might be directly carcinogenic. It is also possible that HCV-induced chronic antigenic stimulation is the primary cause of lymphoma formation [52].

Nearly 90% of individuals with NHL have had cryoglobulinemia, and 35% of patients with B-cell lymphoma have been documented to have HCV viremia [53]. Regarding the existence of HCV in cancerous cells, there are, nevertheless, contradictory data [54,55]. While some studies have shown HCV RNA in bone marrow and lymphoid cells, others have not found HCV in lymphoma cells [55].

The most common NHL types linked to MC are follicular lymphoma (FL), lymphoplasmacytic lymphoma/immunocytoma (LPL), B-cell chronic lymphocytic leukemia/small lymphocytic lymphoma (B-CLL), and marginal zone lymphoma (MZL) [56].

Around 65% of HCV-linked NHL cases affect organs outside lymph nodes, mainly liver and salivary glands, compared to 19% in non-HCV NHL. Meta-analyses reveal a strong link between HCV infection and NHL [57].

A correlation was observed with a relative risk range of 1.8 to 10.8, although lower risk estimates were reported in more recent studies. In countries with high HCV prevalence, such as Italy and Japan, the association was stronger, while in some countries with low HCV prevalence, no association was found [57].

### 3.3. Arthralgia, Myalgia, and Sicca Syndrome

Rheumatologic manifestations are common in chronic HCV infections. Up to 74 percent of patients experience arthralgia and/or arthritis [56]. Joint pain (arthralgia) represents the most frequent extrahepatic symptom in patients with HCV, occurring in approximately 23% of cases—even in those without cryoglobulinemia [58]. In rheumatology, HCV serological testing is recommended in cases with systemic or unexplained symptoms such as vasculitis, sicca syndrome, recent polyarthritis, or fatigue. Among HCV-infected patients, arthritis is often associated with mixed cryoglobulinemia and typically presents as non-erosive polyarthritis [58].

Although less frequent, arthritis not linked to cryoglobulinemia has been acknowledged as a separate clinical form since the mid-1990s [59,60].

According to reports, 40–80% of people infected with HCV develop arthritis [61,62]. The hands and knees are the main joints affected by the symmetrical, non-deforming joint discomfort that patients experience. Arthritis linked to HCV is less prevalent. Joint pain (arthralgia) occurs in nearly 70% of patients experiencing extrahepatic manifestations, particularly associated with type III EMC, commonly involving the proximal interphalangeal and metacarpophalangeal joints, as well as the knees and ankles [63].

Although it is not associated with joint disease, 70–80% of MC patients have rheumatoid factor activity. There are no antibodies against cyclic citrullinated peptide. The clinical appearance of HCV infection might be complicated by the exacerbation of arthralgia and myalgia caused by IFN and other treatment approaches. It is crucial to determine if symptoms such as arthritis, myalgia, and arthralgia that emerge in individuals infected with HCV are caused by a chronic HCV infection or a newly formed rheumatologic illness [64].

Less than 5 percent of individuals with Sjögren’s disease have a positive HCV test, but 20 to 30 percent of patients with HCV infection have sicca symptoms that affect the lips or eyes [65]. “True” Sjögren’s syndrome and HCV-related sicca syndrome share many similarities [66].

### 3.4. Autoantibody Production

Common immunological abnormalities include the following: mixed cryoglobulins (60–90 percent), rheumatoid factor activity (70 percent), antinuclear antibodies (20–40 percent), anticardiolipin antibodies (15 percent), antithyroid antibodies (12 percent), and anti-smooth muscle antibodies (7 percent) [67]. These autoantibodies do not correspond with connective tissue disease symptoms, with the exception of mixed cryoglobulins. B-cell proliferation and HCV-induced hyperactivation are reported as underlying mechanisms.

Between 40 and 65 percent of patients with HCV infection have autoantibodies, including antinuclear antibodies (ANA), RF, anticardiolipin antibodies (aCL), antibodies against thyroid peroxidase, cryoglobulins, anti-smooth muscle, and anti-liver–kidney microsomal antibodies [67,68]. The potential of HCV to affect lymphocytes is the cause of the increased synthesis of autoantibodies [69].

### 3.5. Type 2 Diabetes with Insulin Resistance (IR)

Patients with HCV are 11.5 times more likely than the general population to acquire type 2 diabetes mellitus (T2DM) [70]. Studies confirm a strong link between HCV infection and insulin resistance [71,72]. Insulin resistance and diabetes promote liver fat accumulation, worsening HCV-related disease. Fat accumulation can cause non-alcoholic fatty liver disease, increasing liver damage and fibrosis [73]. The level of insulin resistance was significantly greater among non-obese, non-diabetic people with HCV infection [74], according to a study analyzing IR within HCV-infected patients. This suggests that HCV acts as a distinct variable in inducing IR, regardless of diabetes or overweight status [75,76].

The research on type 2 diabetes supports the idea that an HCV infection raises the likelihood of getting the disease. The chronic nature of HCV infection appears to affect glucose metabolism by altering the host’s innate immune responses [77]. Genotype 1 HCV infection predisposes individuals over the age of 40 to T2DM more frequently than those without HCV [78,79].

HCV-related diabetes is driven by inflammation, liver fat buildup, and insulin resistance. The link between HCV and type 2 diabetes has been supported by studies since the early 1990s [80]. In extensive epidemiological investigations, Caronia et al. [81] and Mason et al. [82] found that diabetes mellitus was more frequently caused by HCV than by HBV-associated liver cirrhosis. The difference was 23.6 percent versus 9.4 percent (OR 3.4, 95% CI 1.6–4.79; *p* = 0.0002), and liver cirrhosis along with male sex were associated with diabetes [80,83].

Multiple mechanisms, including direct viral effects, insulin resistance, proinflammatory cytokines, cytokine signaling suppressors, and other immune-mediated pathways, appear to be involved in the HCV and T2DM association. Insulin resistance and T2DM appear to be primarily caused by the HCV core protein, which is involved in many of these processes. Insulin resistance and type 2 diabetes have been most closely linked to HCV genotypes 1 and 4 [81]. Achieving sustained virologic response in patients with chronic hepatitis C has been consistently associated with a reduced risk of metabolic and vascular complications, including diabetes mellitus. These benefits are likely mediated through improved insulin sensitivity and beta-cell function. Moreover, evidence from large cohort studies, such as the one by Li et al. [84], highlights the broader impact of SVR in significantly lowering the incidence of serious outcomes such as acute coronary syndrome, end-stage renal disease, stroke, and retinopathy. These findings underscore the importance of early antiviral intervention to mitigate long-term systemic complications of HCV infection [52,84].

### 3.6. Cardiovascular Disorders (Carotid Atherosclerosis, Coronary Artery Disease, and Ischemic Heart Disease with Coronary Vasculitis)

Many studies have examined the link between HCV and cardiovascular events like heart attacks and ischemic stroke, congestive heart failure, angina pectoris, and transient ischemic attack. These studies have had different observations, some emphasizing a protective role of HCV, and others identifying it as a risk factor [85,86,87,88,89,90,91,92,93,94,95].

Atherosclerosis is an inflammatory disease that persists over a long period of time. The mechanisms by which HCV may induce or facilitate atherosclerosis remain not fully understood [96,97]. In addition, it is hypothesized that increased levels of pro-atherogenic chemokines and cytokines may activate pro-atherogenic metabolic factors, and, thus, through direct or indirect mechanisms, may induce atherosclerosis [98]. There is evidence that in HCV patients, atherosclerosis is linked to advanced liver fibrosis [99].

The study by Drazilova et al. describes a significant association between chronic hepatitis with HCV and an increased risk of cardiovascular and cerebrovascular disease, both in chronic forms and as an acute event [100].

Huang et al. reported that HCV patients had a higher heart attack risk than uninfected individuals, even with similar cholesterol levels [101]. Additionally, younger patients experienced greater effects than older ones [101].

In 1992, Ishizaka et al. first linked HCV infection to carotid atherosclerosis in a Japanese health screening study [101]. HCV patients had greater intima–media thickness and more carotid plaques (64% vs. 25%) than uninfected individuals. The link between HCV and carotid atherosclerosis remained significant after adjusting for metabolic risk factors [102].

HCV infection has been associated with an increased risk of coronary artery disease, independent of traditional cardiovascular risk factors [86]. Lee et al. [7] found higher cardiovascular mortality (HR 1.50; 95% CI: 1.10–2.03) in anti-HCV-positive individuals, especially those with active viremia, while RNA-negative patients had outcomes similar to the general population. Maruyama et al. [103] reported improved myocardial perfusion in patients achieving SVR, whereas relapsers experienced worsening perfusion following an initial, transient improvement.

A cohort of over 12,800 HCV patients treated with DAAs (compared to untreated patients matched for demographic and clinical characteristics) showed significantly lower mortality from extrahepatic complications, including cardiovascular disease, chronic kidney disease, diabetes, and mental health disorders [104]. Successful treatment (achieving SVR) was associated with an approximately 56% reduction in extrahepatic mortality [104].

### 3.7. Renal Insufficiency and Glomerulonephritis

HCV uses CD81 and SR-B1 receptors to infect kidney cells [105]. HCV RNA has been found in various kidney cell types, including mesangial, tubular, and capillary endothelial cells [106,107]. Higher levels of proteinuria have been linked to granular HCV-related protein deposits in the mesangium [108].

Approximately 40% of HCV patients develop extrahepatic symptoms as the illness worsens [65]. It is also commonly recognized that proteinuria is widely linked with HCV infection [109]. Post-transplant HCV infection affects kidney graft success and long-term patient survival [110].

A large study found that HCV patients had a higher risk of end-stage renal disease (4.3 per 1000 person-years) than uninfected individuals, according to a retrospective cohort study involving approximately 470,000 individuals [111]. According to this new study, patients with HCV seropositive status were 40 percent more likely than those with seronegative status to develop renal insufficiency, which is defined as blood creatinine levels of at least 1.5 mg/dL [112].

### 3.8. Porphyria Cutanea Tarda

HCV infection is common among patients with porphyria, with rates ranging from 40 to 50 percent depending on the country [112,113]. This association is due to decreased hepatic activity of uroporphyrinogen decarboxylase. As a result, there is overproduction and accumulation of uroporphyrinogen compounds in the blood and urine of affected patients.

Photosensitivity, skin fragility, bruising, and potentially hemorrhagic vesicles or bullae are among the clinical characteristics. Skin thickening, hirsutism, baldness, and hypo- or hyperpigmentation are examples of chronic findings [112].

### 3.9. Lichen Planus

The recurrent pruritic eruption known as lichen planus (LP) is characterized by the appearance of flat-topped, violaceous papules that can occur on any part of the skin, such as the arms, trunk, genitals, nails, and scalp, as well as on oral mucous membranes [113,114].

Regarding the association between LP and HCV, a number of epidemiological studies have shown that HCV is prevalent among individuals with LP (22.3 percent), although some geographic variability exists. Studies investigating the pathogenic mechanisms suggest that LP is not caused by the virus itself but rather by a host immune response to HCV antigens [115].

### 3.10. Thyroid Disorders

The most common thyroid pathology encountered in HCV patients is autoimmune thyroid disease [116]. The pathophysiological hypothesis includes a T-cell-mediated autoimmunity. Circulating thyroid autoantibodies are detected in 80–85% of cases; hypothyroidism is observed in approximately 30% of patients [7]. A meta-analysis notes that hypothyroidism, the presence of anti-thyroglobulin antibodies, anti-thyroid microsomal antibodies, and anti-thyroid peroxidase antibodies are higher in HCV patients than in controls [117].

Papillary thyroid cancer has also been observed more frequently in patients with chronic HCV hepatitis, especially in those with autoimmune thyroid disease [115]. In a recent study, a history of HCV infection was associated with an increased risk of thyroid cancer [118].

### 3.11. Neuropsychiatric Disorders

Patients with chronic HCV infection present with various neuropsychiatric disorders [119]. Depression is more common in HCV patients than in the general population (59% vs. 21%) [118]. Sleep disorders have been observed in approximately 60% of HCV-infected patients [119], and fatigue in 50–67% of them [120]. Mood disorders [121,122], as well as cognitive impairment [123,124,125], are more frequent in HCV patients than in the general population. Pathophysiological hypotheses include viral entry into the central nervous system and infection of peripheral CD68+ cells [123]; viral proteins may have neurotoxic effects [126,127] and HCV may directly affect serotonergic and dopaminergic neurotransmission with possible depressive symptoms [128]. Current evidence suggests that HCV infection may contribute to the development of neuropsychiatric manifestations through multiple mechanisms. These include chronic immune activation, elevated levels of proinflammatory cytokines such as interleukin-6 (IL-6) and tumor necrosis factor-alpha (TNF-α) [80,98,129], as well as possible viral neuroinvasion. Additionally, the HCV core protein has been associated with disturbances in glucose and lipid metabolism, promoting insulin resistance through interference with IRS-1/PI3K signaling pathways, which may indirectly contribute to cognitive decline and affective disorders [130,131].

### 3.12. Study Limitations

A first limitation of the study is that, being a narrative analysis performed on a small number of studies that met the inclusion criteria, a consistent quantitative analysis and meta-analysis are lacking. However, this analysis brings comparative and summarized information that is extremely important for clinical practice in an interdisciplinary, understudied field, namely that of extrahepatic manifestations induced by HCV.

However, heterogeneity in study design, patient populations, treatment duration, and therapeutic regimens among the included studies may limit the generalizability of these findings.

## 4. Conclusions

The emergence of new interferon-free DAA treatments, which are both safe and highly effective in eliminating HCV in nearly all cases, has transformed the outlook for these patients. HCV-associated extrahepatic consequences are less common and less severe when HCV is eliminated with DAAs, according to several studies. It has been discovered that DAA-assisted HCV eradication considerably enhances cardiovascular health and lowers the incidence of MC cases. As a result, a decrease in MC cases linked to HCV is anticipated in the upcoming years.

Additionally, early DAA initiation prevents irreversible extrahepatic damage. Future studies should explore long-term outcomes post-SVR in high-risk subgroups, such as patients with cryoglobulinemia.

A prompt treatment strategy not only facilitates the resolution of numerous extrahepatic manifestations that remain in a reversible phase of the disease but also helps prevent a considerable number of extrahepatic conditions that may arise due to delayed treatment.

## Figures and Tables

**Table 1:** Extrahepatic manifestations associated with HCV infection.

Category	Associated Conditions
Immune	Mixed cryoglobulinemia
	Non-Hodgkin lymphoma
	Joint and muscle pain (arthralgia/myalgia)
	Sicca syndrome (similar to Sjögren’s)
	Polyarteritis nodosa
	Monoclonal gammopathy
	Presence of autoantibodies
Inflammatory/Metabolic/Vascular	Glomerulonephritis
	Renal dysfunction
	Insulin resistance
	Type 2 diabetes mellitus
	Ischemic heart disease, potentially with coronary vasculitis
	Carotid artery atherosclerosis
	Coronary artery disease
Dermatological	Lichen planus Porphyria cutanea tarda
Thyroid	Autoimmune thyroid disease
	Hypothyroidism
	Thyroid cancer
Neuropsychiatric	Mood disorders (including depression)
	Cognitive decline
	Sleep disorders
	Fatigue

**Table 3:** DAA therapy in HCV-associated mixed cryoglobulinemia.

Author	Type of Study	No. of Patients	Age (Mean, Range)	Baseline HCV-RNA (log_10_ IU/mL)	ALT (IU/L)	Purpura n (%)	Arthralgia n (%)	Polyneuropathy n (%)	Skin Ulcer n (%)	Renal Involvement n (%)	DAA Regimen	Duration (Weeks)	Virological Response (SVR)	Clinical Response
Gragnani et al., 2016 [25]	Prospective	44	65.3 ± 10.1	2.9 ± 3.6	77.7 ± 77.2	32 (73)	26 (59)	28 (63)	6 (14)	4 (9)	SOF + RBV 24: 18 (41%) SOF + SIM (+RBV): 12 (27%) SOF + DAC (+RBV): 4 (32%) SOF + LED (+RBV): 10 (23%)	12 or 24	100%	CR: 66% PR: 42% NR: 7%
Saadoun et al., 2017 [19]	Prospective	41	56 (50–62)	5.9 ± 0.2	55.3 ± 6.4	31 (75.6)	26 (63.4)	21 (51.2)	7 (17.1)	5 (12.2)	SOF + DAC: 32 (78%) SOF + DAC: 9 (22%)	12 or 24	100%	CR: 90% PR: 4% NR: 0
Lauletta et al., 2017 [33]	Prospective	22	66.9 ± 11.2 (46–84)	6.02 ± 1.2	104.8 ± 144.7	12 (55)	12 (55)	10 (45)	NA	NA	3D: 3 (14%) SOF + RBV: 10 (45%) SOF + SIM ± RBV: 4 (18%) SOF + LED ± RBV: 5 (23%)	12 or 24	100%	CR: 64% PR: 23% NR: 14%
Emery et al., 2017 [42]	Retrospective	18 symptomatic 65 asymptomatic 10 severe	58	NA	NA	15 (83.3)	NA	6 (33)	NA	10 (55)	IFN/RBV/DAA: 7 (39%) IFN-free: 11 (61%) SOF + RBV: 5 (28%) SOF + SIM: 3 (28%) SOF + LED ± RBV: 3 (16%) 3D + RBV: 2 (11%)	12 or 24	88.9% (symptomatic), 90.8% (asymptomatic)	CR: 39% PR: 22% NR: 39%
Passerini et al., 2018 [41]	Prospective	35	67 (12.7)	4.72–5.85	46 (27–84)	24 (69)	6 (17)	11 (31)	NA	2 (6)	SOF-based regimen: 31 (88%) 2D regimen: 1 (9%) 3D regimen: 3 (8%)	12 or 24	100%	CR: 43% PR: 37% NR: 20%
Bonacci et al., 2018 [32]	Retrospective	46	61 (53–01)	5.9 (5.4–6.2)	65 (34–119)	29 (63)	16 (35)	19 (41)	NA	9 (20)	SOF-based regimen: 21 (46%) 3D regimen: 13 (28%) SIM + DAC: 4 (9%) GZB + EBR: 3 (6%) PegIFN + DAAs: 4 (9%) FDV + LDR: 1 (2%)	12 or 24	100%	CR: 80% PR: 11% NR: 9%
Mazzaro et al., 2018 [40]	Retrospective	22	69 (39–74)	5.80 (4.31–7.00)	72 (12–173)	12 (55)	12 (55)	10 (45)	NA	NA	3D: 3 (14%) SOF + RBV: 10 (45%) SOF + SIM ± RBV: 4 (18%) SOF + LED ± RBV: 5 (23%)	12 or 24	95%	CR: 64% PR: 14% NR: 23%
Cacoub et al., 2019 [27]	Prospective	148	57 (51–67)	5.3 ± 6.5	24 ± 77	85 (57.4)	94 (64.4)	86 (58.1)	15 (10.1)	25 (16.9)	SOF + RBV: 51 (34%) SOF + DAC: 53 (36%) SOF + LED: 23 (16%) SOF + SIM: 18 (12%)	12 or 24	95.2%	CR: 72% PR: 22% NR: 5%
Pozzato et al., 2020 [43]	Retrospective	67	NA	NA	NA	33 (49)	36 (54)	28 (42)	NA	NA	SOF-based: 52 (78%) 3D regimen: 12 (18%), Asunaprevir/DAC: 4 (6%)	12	95%	CR: 60% NR: 40%

Abbreviations used: ALT: alanine aminotransferase; HCV-RNA: hepatitis C virus ribonucleic acid; DAA: direct-acting antiviral; SOF: sofosbuvir; DAC: daclatasvir; RBV: ribavirin; LED: ledipasvir; SIM: simeprevir; 3D: ombitasvir/paritaprevir/ritonavir + dasabuvir; 2D regimen: ombitasvir/paritaprevir/ritonavir; IFN: interferon; FDV: faldaprevir; LDR: deleobuvir; GZB: grazoprevir; EBR: elbasvir; NA: not available; SVR: sustained virological response; CR: complete response; PR: partial response; NR: no response.

## Data Availability

Data availability statements are available upon request through the corresponding author.
